# Measurement of Noradrenaline and Serotonin Metabolites With Internal Jugular Vein Sampling: An Indicator of Brain Monoamine Turnover in Depressive Illness and Panic Disorder

**DOI:** 10.3389/fpsyt.2022.818012

**Published:** 2022-06-02

**Authors:** Murray Esler, Marlies Alvarenga, David Barton, Garry Jennings, David Kaye, Ling Guo, Rosemary Schwarz, Gavin Lambert

**Affiliations:** Human Neurotransmitter Research Laboratory, Baker Heart and Diabetes Institute, Melbourne, VIC, Australia

**Keywords:** brain serotonin turnover, brain noradrenaline turnover, sympathetic nervous system, cerebral venous sinuses, panic disorder, depressive illness

## Abstract

In research spanning three decades we have estimated brain monoamine turnover (approximately equating with synthesis rate) with sampling from the internal jugular veins and measurement of trans-cerebral plasma monoamine metabolite concentration gradients. Here we report indices of brain noradrenaline and serotonin turnover in patients with major depressive illness (MDD) and panic disorder (PD). Brain noradrenaline turnover was assessed from the combined flux into the internal jugular veins of the metabolites dihydroxyphenylglycol (DHPG) and 3-hydroxy-4-methoxyphenylglycol (MHPG), and brain serotonin turnover from the overflow of the primary metabolite, 5-hydroxyindole acetic acid (5HIAA). Comparison was made with matched healthy research participants. In both MD and PD the estimate of brain noradrenaline turnover provided by metabolite overflow was unremarkable. In contrast, in both patient groups the estimate of brain serotonin turnover provided by 5HIAA overflow was increased 3–4-fold (*P* < 0.01). This neurotransmitter abnormality was normalized in MDD and PD in clinical remission, during selective serotonin reuptake blocker (SSRI) dosing. We cannot be sure if the brain serotonergic abnormality we find in MDD and PD is causal or a correlate. Measurements in PD were not made during a panic attack. The increased estimated serotonin turnover here may possibly be a substrate for panic attacks; serotonergic raphe nuclei participate in anxiety responses in experimental animals. It is puzzling that the findings were identical in MDD and PD, although it may be pertinent that these psychiatric diagnoses are commonly comorbid. It is unlikely that activation of brain serotonergic neurons is driving the sympathetic nervous activation present, which contributes to cardiovascular risk, persistent sympathetic activation in MDD and episodic activation in PD during panic attacks. We have previously demonstrated that the mechanism of activation of human central sympathetic outflow in other contexts (hypertension, heart failure) is activation of noradrenergic brainstem neurons projecting to the hypothalamus and amygdala.

## Introduction

The brain neurotransmitter changes characterizing psychiatric disorders are uncertain. Reserpine, a now almost obsolete antihypertensive drug, which depletes the brain of noradrenaline, quite commonly caused depressive illness, observations which Schildkraut drew on in the formulation of his historic noradrenaline deficiency hypothesis of depression causation (1). More recently, it has been a common practice to infer brain neurochemical mechanisms of psychiatric illness from the known pharmacological actions of efficacious psychotropic drugs. Tricyclic antidepressant drugs are neuronal noradrenaline reuptake-blockers. The efficacy of this drug class has sometimes been taken to directly derive from augmentation of CNS noradrenaline neurotransmission, by transmitter uptake blockade correcting the deficiency hypothesized by Schildkraut (1). Analogous thinking saw the clinical benefit of selective serotonin reuptake blocking drugs in depressive illness and panic disorder attributed to a correction of an existing “serotonin neurotransmission deficiency” (2). This line of thinking although provocative is imprecise. There is a need to evaluate noradrenergic and serotonergic neuronal systems directly in the human brain in depressive illness and panic disorder. This, however, has proven difficult. More commonly, monoamine chemistry has been studied outside the brain, as a surrogate for CNS neurochemistry.

## Investigation of Monoamine Chemistry Outside the Brain as a Surrogate for CNS Noradrenaline and Serotonin Neurochemistry

It turns out that this widely adopted approach is rather dubious. Peripheral monoamine chemistry casts a pale shadow of brain neurochemistry. The brain is the source of <10% of the major serotonin metabolite, 5-hydroxyindole acetic acid (5-HIAA) found in plasma and urine (3) rendering systemic plasma measurements and urine collections unsuitable for studying brain serotonin release. Further, serotonin sampled in plasma mainly originates from platelets activated in the blood sampling process. With lumbar CSF, the serotonin is derived more from the terminals of descending serotonergic neuronal projections to the spinal cord than from the brain (4). For a time, measurement of the deaminated methylated metabolite of noradrenaline, MHPG, in plasma and urine was proposed and widely used as an index of brain noradrenaline turnover. This was not valid. Our subsequent investigation of regional inputs of MHPG to the plasma pool demonstrated that no more than 10% was derived from the brain, the primary source being noradrenaline contained in and released from sympathetic nerves (5).

## Assessing Brain Monoamine Turnover Using Internal Jugular Vein Sampling

**Figure 1:**
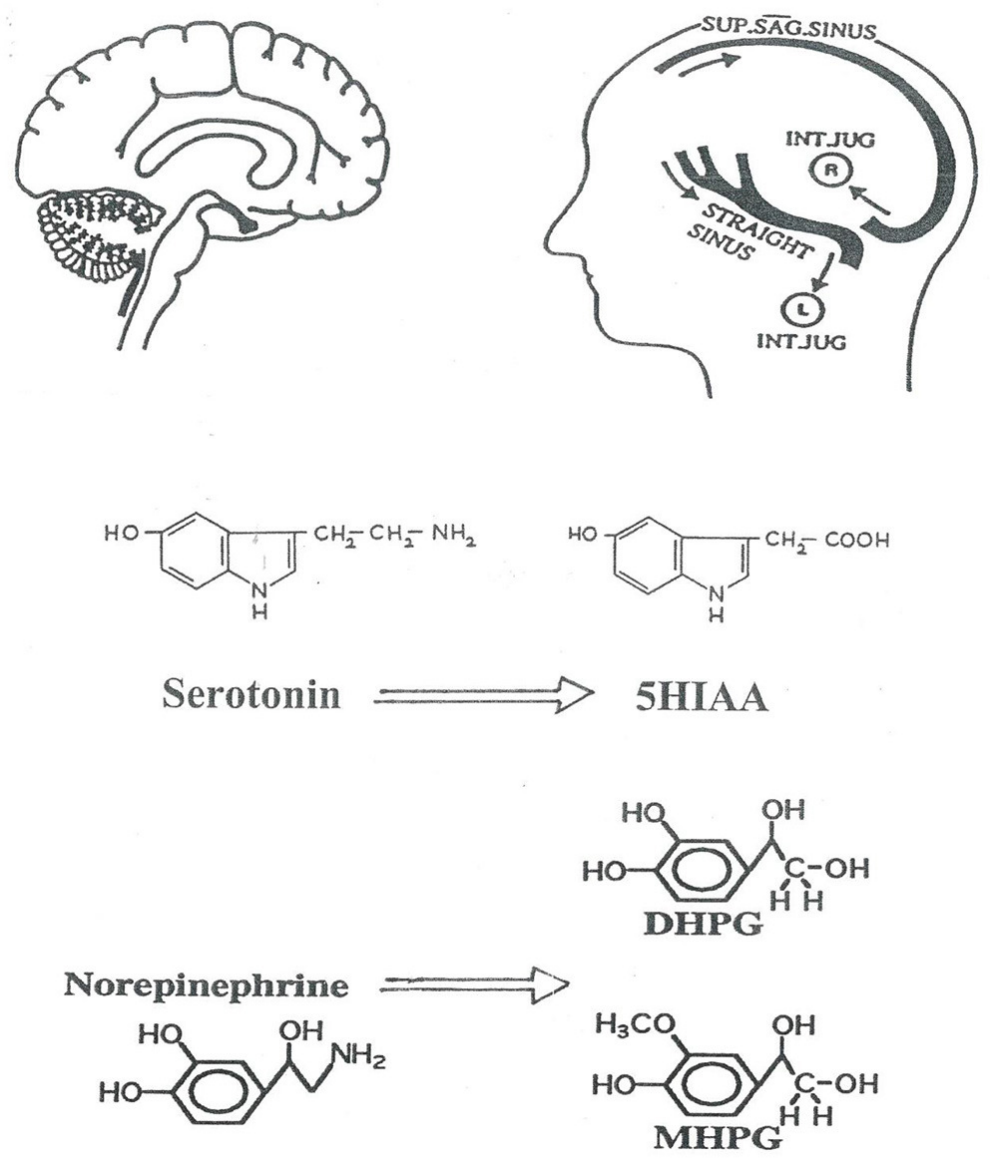
Schematic representation of the investigation of human brain monoamine turnover, utilizing internal jugular venous sampling and the assay or serotonin and noradrenaline metabolites in plasma, to measure jugular venous—arterial plasma concentration differences. 5HIAA, 5-hydroxyindoleacetic acid is the principal metabolite of serotonin; DHPG, dihydroxyphenylglycol; MHPG, 3-methoxy-4-hydroxyphenylglycol are lipophilic metabolites of norepinephrine which cross the blood-brain barrier. The lateralization of cerebral venous sinus drainage is represented, explained in Figure 2. SUP. SAG. SINUS, Superior Sagittal Sinus; INT. JUG., internal jugular vein.

The Human Neurotransmitters Research Laboratory of the Baker Institute in Melbourne, following the lead of James Maas working with non-human primates (6) developed techniques for assessing brain monoamine turnover in human illness based on jugular venous sampling of monoamine metabolites (7–10). CNS monoamine turnover approximately equates with monoamine synthesis rates. This is certainly not a direct measure of neurotransmitter release (11). The predominant determinant of turnover is cytosolic metabolism of monoamines released from stored vesicles (11). Neurotransmitter release, however, does contribute to monoamine turnover. We use brain monoamine metabolite overflow as a semiquantitative index of neurotransmitter release. As applied here in the assessment of brain noradrenaline and serotonin turnover in MDD and PD (Figure 1), the technique involves measuring the overflow of metabolites of noradrenaline (MHPG, 3-methoxy-4-hydroxyphenylglycol; DHPG dihydroxyphenylglycol) and serotonin (5-hydroxyindoleacetic acid) into the internal jugular veins (3, 4, 8–10). Simultaneous arterial and jugular venous blood samples are obtained. Brain noradrenaline and serotonin turnover can be assessed, using the Fick principle, from venous-arterial plasma concentration differences and thermodilution measurements of internal jugular blood or plasma flows (8–10). For applying the Fick principle the preferred flow measurement differs for individual metabolites (3, 4).

**Figure 2:**
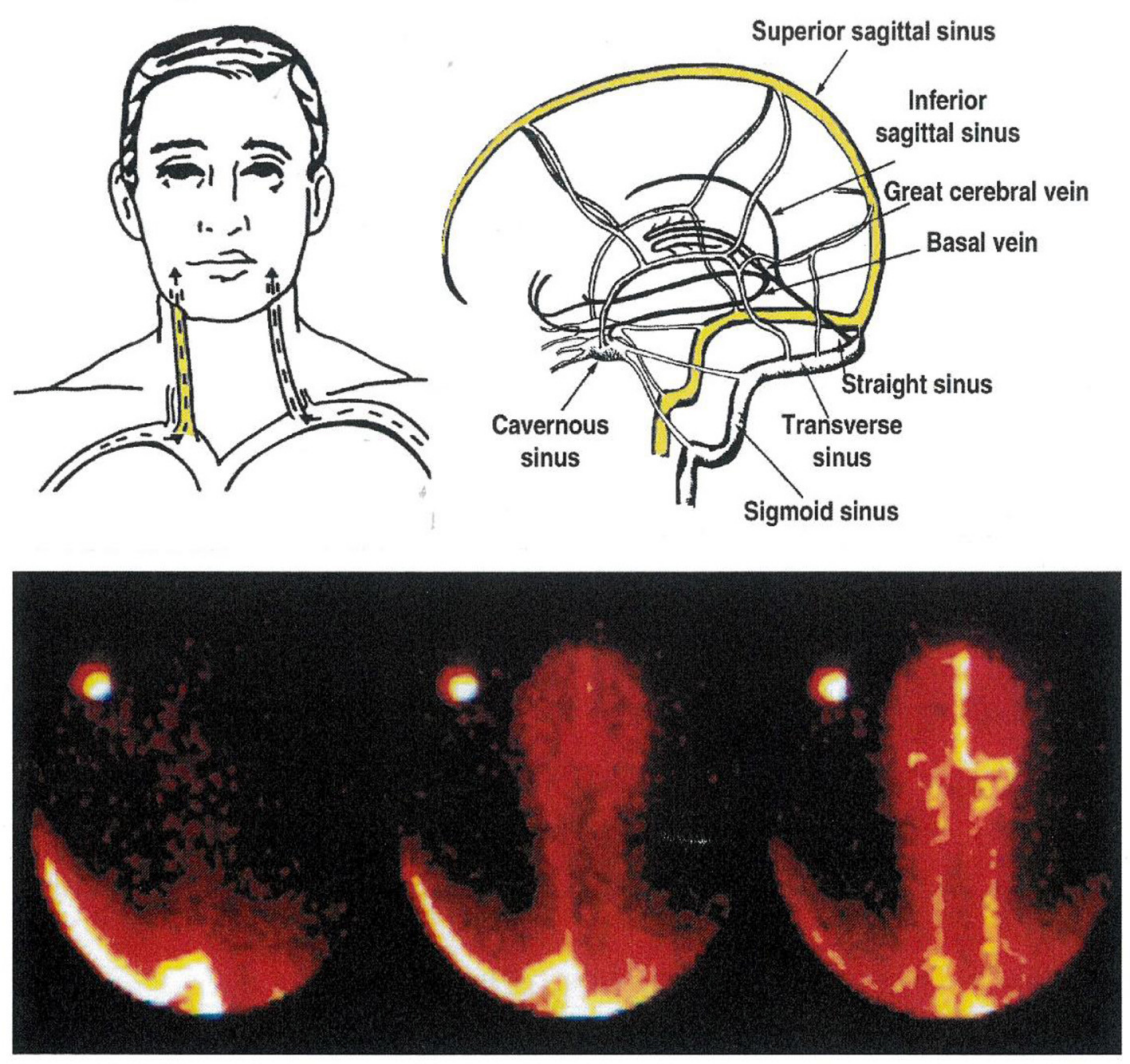
The cerebral venous sinuses of the brain. The lower panel is of a Technetium-99 scan of the sinuses, recorded from behind. In this patient, the sagittal sinus drains to the right, into the transverse sinus and the right internal jugular vein. The straight sinus, with flow from the hypothalamus and amydala in particular, drains into the left internal jugular vein. This is the commonest anatomical pattern, but is reversed in some people. Knowledge of the pattern of brain venous drainage in individual patients, with bilateral internal jugular vein sampling, allows selective quantification of monoamine turnover in cortical, and in suprabulbar subcortical brain regions. The brainstem drains into the spinal veins.

Existing asymmetry in human cerebral venous drainage (Figure 2) can be drawn on to study the regional CNS origins of monoamine metabolites (8, 12). The right internal jugular vein typically has the superior sagittal sinus as its major tributary, and accordingly drains the cerebral cortex (Figure 2). In contrast, the left internal jugular vein typically is a continuation of the straight sinus and drains suprabulbar, subcortical brain regions (8, 12). This lateralized pattern of flow is occasionally reversed. Performing a Technetium 99 single photon emission computed tomographic (SPECT) scan of the brain venous sinuses (8, 10) allows identification of the drainage pattern (Figure 2). Further, venous scanning demonstrates that typically, although not invariably the two drainage pathways are distinct, with little or no blood admixture at the confluence of the sinuses (Figure 2) (8, 10).

The hypothesis tested was that brain neuronal noradrenergic and serotonergic activity is low in untreated patients with MDD and PD. Here we report our findings in two clinical data sets, in untreated patients with major depressive disorder (MDD) and panic disorder (PD). These patient groups were selected to minimize comorbidity, and described separately previously (10, 13). Because of the remarkable and unexpected similarities in the findings in both disorders they are analyzed together in this manuscript.

## Methods

Brain monoamine metabolite overflow in major depressive disorder, and in panic disorder was studied with arterial and internal jugular venous blood sampling as previously reported (3, 4, 8, 10, 13) and described above. High internal jugular venous sampling was used, with the catheter tip placed beyond the angle of the jaw to exclude sampling from the venous drainage of the face. The central venous catheter, a 7F coronary sinus thermodilution catheter (Webster laboratories, type CCS-7U-90B) introduced *via* an antecubital venous sheath, was percutaneously inserted under local anesthesia, and placed with fluoroscopic control high in one internal jugular vein, or both if this could be achieved without difficulty. Measurement of internal jugular blood flow by thermodilution using the Webster catheter (8, 10) was performed in the panic disorder patients. In MDD patients, jugular flow measurements could not be done, as just prior to the commencement of the study the Webster catheter became unavailable. Arteriovenous blood and plasma concentration differences of the metabolites across the brain, without flow measurements, were used.

### Panic Disorder

Thirty-four patients meeting DSM IV (14) diagnostic criteria for panic disorder, who were either never treated or had received no medication in the preceding 3 months, and 24 healthy volunteers participated. The PD patients and healthy participants were of similar age (Table 1). Clinical evaluation was independently performed by a clinical psychologist and psychiatrist participating in the study. After initial screening, a structured clinical interview based on DSM-IV was used to make a clinical diagnosis of panic disorder (14). Patient selection attempted to minimize psychiatric comorbidity. Fourteen panic disorder patients had a secondary diagnosis of depression. Assessment of overall panic disorder severity was obtained using the patient self-rating Panic Disorder Severity Scale (PDSS) (16) and with a clinician-applied scale, ADIS-IV (17).

**Table 1:** Brain 5HIAA overflow in panic disorder.

	**Panic disorder**	**Healthy subjects**
Age	43.2 (2)	44 (1)
BMI	23.7 (0.7)	23.4 (0.6)
Bright Sunlight (hours/day)	5.7 (0.8)	5.0 (0.9)
Jugular vein blood flow (bilateral - ml/min)	994 (70)	868 (64)
Arterial plasma 5-HIAA conc. (pmol/ml)	32.7 (1.3)	40.8 (4.2)
Jugular vein plasma 5-HIAA conc. (pmol/ml)	35.9 (1.4)	41.5 (4.2)
Brain 5-HIAA overflow(pmol/min)	1,815 (223)^**^	361 (73)

*Listed jugular blood flow represents the sum of bilateral measurements, or doubled unilateral measurement when only this is available. Bright sunlight hours on the study day was provided by the Melbourne Bureau of Meteorology (15)*.

** *P < 0.01 (Mann Whitney rank sum test)*.

*Means and standard errors are listed*.

*BMI, body mass index; 5-HIAA, 5-hydroxyindoleacetic acid*.

### Major Depressive Disorder

Twenty-one patients fulfilling DSM IV diagnostic criteria for major depressive disorder and 40 healthy volunteers participated. Patients were either newly diagnosed or currently untreated after relapse and had not been receiving antidepressant medication for at least 4 weeks prior to the study. The MDD patients and healthy participants were of similar age (Table 2). After initial screening, patients were interviewed by a psychiatrist using a structured clinical interview, the Mini International Neuropsychiatric Interview (MINI) (18). The 17-item Hamilton Depression Scale (HAM-D) (19), Spielberger's State and Trait Anxiety Inventories (20, 21), and the Beck Depression Inventory (BDI) (22) were used to assess severity. Patients had HAM-D and BDI scores of 18 or higher, and were assessed as having MDD as the primary illness at Psychiatric interview. Patient selection attempted to minimize recruitment of patients with psychiatric comorbidity. Those assessed to have high suicide risk were excluded. The initial research studies were performed within 10 days of confirmed diagnosis of MDD.

**Table 2:** Estimated brain 5HIAA overflow in depressive illness.

	**Depressive illness**	**Healthy subjects**
Age	45 (1)	41 (1)
BMI	25 (1)	24 (1)
Bright Sunlight (hours/day)	6.6 (0.8)	6.0 (0.9)
Arterial plasma 5-HIAA conc. (pmol/ml)	46 (5)	42 (4)
Arterial - Jug. vein plasma 5-HIAA conc. difference (pmol/ml)	4.4 (0.9)^**^	1.6 (0.5)

*Listed bright sunlight hours on the study day was provided by the Melbourne Bureau of Meteorology (15)*.

** *P < 0.01 (Mann Whitney rank sum test)*.

*Means and standard errors are listed*.

*BMI, body mass index; 5-HIAA, 5-hydroxyindoleacetic acid*.

#### Avoidance of Potential Confounding by Season, Sunlight and Adiposity

Brain serotonin releasing neurons subserve diverse, although incompletely understood functions related to feeding and adiposity (23) and to light stimulation (24). We have previously demonstrated an influence of these on brain 5HIAA overflow in humans (15, 25). By appropriate selection and matching of healthy reference populations, with matching of healthy subjects and patients in terms of body mass index and sunlight hours on the study day, we were able to avoid a confounding effect of serotonergic neuronal systems activated by sunlight and in obesity. Mean body mass index was 24–26 kg/m^2^ for the four study groups. With the assistance of the Melbourne Bureau of Meteorology (25) the two healthy volunteer groups were chosen from a large pool of subjects studied by the laboratory such as to achieve matching for sunlight hours on the day of the catheter sampling study, with mean sunlight hours being 5–6 h for the four participant groups (12, 13).

#### Selective Serotonin Reuptake Inhibitor Dosing

In both patient groups, after the initial investigational study, testing was performed for an influence of SSRI dosing on brain 5HIAA generation. This was done open-label in 11 patients with PD, using citalopram at a dose of 20–60 mg daily, given for a minimum of 3 months, followed by a repeat investigational study. Dosage was determined by clinical response. Similarly, 11 patients with MDD were treated with differing SSRIs, the choice of drug being made on clinical grounds, for ~12 weeks, followed by the second investigational study. The patients with MDD were followed weekly for the purposes of the study, or more frequently if required on clinical grounds.

#### Monoamine Plasma Assays, Brain Monoamine Turnover Estimation

Jugular-arterial venous-arterial plasma concentration differences of MHPG, DHPG and 5-HIAA were measured, with sampling from an internal jugular catheter and from an indwelling radial or brachial artery cannula. In patients with MDD and the matching reference population of healthy volunteers only the transcerebral metabolite plasma concentration differences were available. In patients with PD and matched healthy participants brain noradrenaline and serotonin turnover were assessed using the Fick principle, from the venous-arterial plasma concentration differences and from internal jugular flows, measured by thermodilution (3, 4, 10, 13). Because of differing equilibration of monoamine metabolites between blood and plasma (fast for MHPG and DHPG, slow for 5-HIAA), the turnover calculation used blood flow for MHPG and DHPG, and plasma flow for 5-HIAA (3, 5). Measurements were made at quiet rest, and in the absence of a panic attack in PD patients. Assay of DHPG, MHPG and 5-HIAA was by high performance liquid chromatography, using previously described methods (3, 4).

#### Statistics

All results, unless otherwise specified, are expressed as mean (standard error of mean). Tests of significance were carried out using ANOVA, paired *t*-tests or distribution-free non-parametric tests where appropriate. The possible relation between variables was evaluated using least squares linear regression analysis. All statistical tests were two-tailed and statistical significance was set at a probability level of 0.05.

#### Research Ethics

The research procedure was performed by cardiologists who are expert in the technique. The process of written consent, to which an honest, open and explicit participant information sheet was central, conformed to the standards expected to preserve the autonomy of the participants. The research was approved by the Alfred Hospital Medical Research Ethics Committee.

## Results

Brain 5HIAA overflow in humans is higher with overweight (15), and with longer periods of intense sunlight exposure (25). For both the PD and MDD patient cohorts, good matching for these variables was achieved with the two comparator groups of healthy participants, to avoid potential confounding (Tables 1, 2) (10, 13).

### Panic Disorder

In panic disorder patients the brain noradrenaline turnover biomarker, internal jugular venous overflow of MHPG plus DHPG, was 1,454 pmol/min, SEM 142, similar to that in healthy participants, 1,336 pmol/min, SEM 77. These values were calculated with bilateral jugular venous sampling, or from doubling the combined unilateral jugular venous overflow of MHPG plus DHPG (findings in volunteers having bilateral sampling justified this adjustment). In contrast, the brain serotonin turnover biomarker (jugular venous overflow of 5HIAA), was increased more than 4-fold in panic disorder, 1,815 pmol/min, SEM 424 compared with 361 pmol/min, SEM 73 in healthy subjects (*P* < 0.01). The arterial plasma 5-HIAA concentrations was not elevated in panic disorder; the brain contributes only 8% to the central plasma pool of 5-HIAA (3). The venoarterial plasma concentration step-up across the brain, however, was higher (Table 1). Severity of panic disorder, scored with the patient self-rating Panic Disorder Severity Scale was strongly and directly correlated with brain 5HIAA overflow; *r* = 0.86, *P* < 0.001. To this point jugular venous sampling has not been performed during a panic attack.

#### Regionalized Brain 5HIAA Overflow in Panic Disorder

In those participants in whom the cerebral sinus venous scan demonstrated lateralized drainage into the internal jugular veins it was possible to compare 5HIAA overflow separately in cortical and subcortical regions. In panic disorder patients this was similarly increased (~4-fold) in cortical and subcortical brain regions. Cortical 5HIAA overflow (14 values in panic disorder, 10 in healthy subjects) was 863 pmol/min, SEM 131 in panic disorder patients and 225 pmol/min, SEM 73 in healthy volunteers (*P* < 0.001; student's *t*-test). Subcortical 5HIAA overflow (nine in panic disorder, seven values in healthy subjects) was 821 pmol/min, SEM 152 in panic disorder patients and 199 pmol/min, SEM 73 in healthy volunteers (*P* = 0.005; student's *t*-test).

### Depressive Illness

Patients with MDD were moderately to severely depressed with a HAM-D score of 25 (1), mean (SE). The BDI score was 29 (2). State anxiety score 57 (3) and trait anxiety score 62 (2) were also elevated. Seven of 21 MDD patients had comorbid (secondary) panic disorder.

In patients with depressive illness, the brain noradrenaline turnover biomarker, estimated from the internal jugular vein—arterial plasma concentration differences of MHPG and DHPG as for PD, was normal, almost identical with the concentration differences present in the healthy participants. The arterial 5-HIAA plasma concentration was similar in MDD and healthy participants, 46 (5) pmol/ml compared with 42 (4) pmol/ml (Table 2) (difference not statistically significant). The internal jugular vein—arterial plasma 5-HIAA plasma concentration difference was 4.4 (0.9) pmol/ml, compared with 1.6 (0.54) pmol/ml in healthy participants (*P* = 0.003), broadly indicative of a probable increase in brain serotonin turnover The 5-HIAA concentration gradient in MDD was not quantitatively linked to the assessed risk of suicide (*P* = 0.54), or to the severity of depression based on the HAM-D rating (*P* = 0.12). The presence of comorbid, secondary panic disorder did not influence estimated brain serotonin turnover in MDD.

#### Effects of SSRI Dosing in PD and MDD

**Figure 3:**
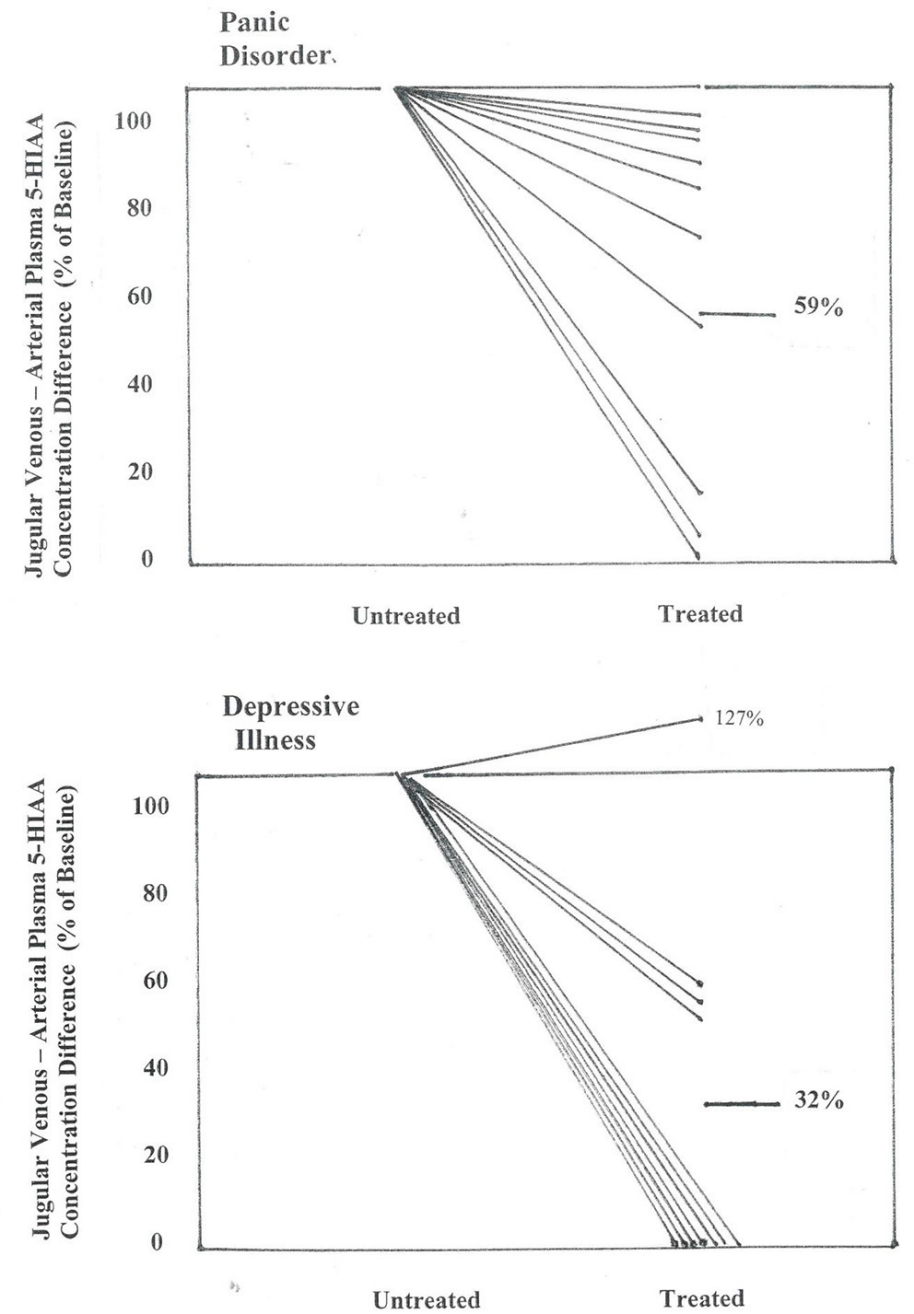
Effect of SSRI dosing on jugular venous—arterial plasma 5-HIAA concentration difference in patients with panic disorder, and with depressive illness. Concentration differences are shown between pre-treatment baseline and after 2–3 months of SSRI administration, when clinical remission had been achieved in the majority of patients. In panic disorder patients the jugular venous—arterial plasma 5-HIAA concentration difference fell to a mean of 59% of baseline (*P* < 0.01), and in patients with MDD, to 32% of baseline (*P* < 0.01) (repeated measures analysis of variance).

In the 10 patients with panic disorder studied before and during SSRI dosing with citalopram, the transcerebral 5-HIAA plasma concentration difference, which while untreated was markedly higher than in normal volunteers, was reduced in the treatment phase, falling in nine of 10 patients, and into the normal range in 5 (*P* < 0.01) (Figure 3). This coincided with clinical improvement. Thermodilution jugular venous flow measurements were not available for the treatment group for direct measurement of brain metabolite overflow.

In the 11 patients with MDD where matching brain monoamine metabolite measurements were available after SSRI dosing, improvement in clinician- and patient-rated symptoms were noted. In this subgroup HAM-D fell to 7 (1), *P* < 0.001, and BDI to 9 (1), *P* < 0.001. The brain 5HIAA plasma concentration gradient was substantially reduced in all but one patient (Figure 3), with a fall from 6.0 (0.9) pmol/ml before treatment to 2.0 (0.7) following SSRI dosing (*P* = 0.008).

#### Relation of Estimated Brain Serotonin Turnover to Sympathetic Nervous System Activation in MDD and PD?

**Figure 4:**
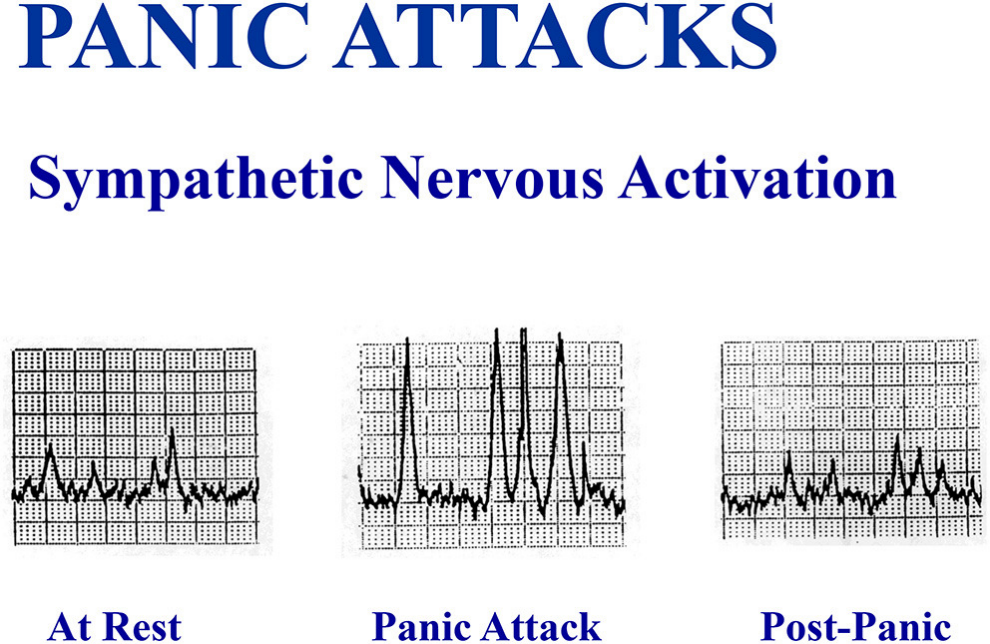
A spontaneous panic attack in the laboratory in an unmedicated patient was accompanied by a large increase in multiunit sympathetic burst size recorded in sympathetic fibers passing to the skeletal muscle vasculature of the leg, without an increase in sympathetic burst firing rate.

Could increased brain serotonin release be the driver of the sympathetic nervous system activation known to be present (26) in these two psychiatric disorders, persistent sympathetic activation in MDD (26, 27) and episodic sympathetic activation in PD, during panic attacks (27, 28)? We have been able to document that high level sympathetic nervous activation occurs during panic attacks (Figure 4), but we have not been able to test whether brain serotonergic activation is the mechanism. Persisting sympathetic activation is a characteristic of MDD. In this study population of patients with MDD, the increase in transcerebral 5HIAA plasma concentration gradient was found to be quantitatively unrelated to the ongoing sympathetic nervous system activation measured with whole body noradrenaline spillover methodology and nerve recording in the sympathetic outflow to the skeletal muscle vasculature (28).

## Discussion

With direct internal jugular venous sampling we found no abnormality in transcerebral noradrenaline plasma metabolite concentration gradient in depressive illness, in conflict with the Schildkraut CNS noradrenaline deficiency hypothesis (1). Similarly in panic disorder, overflow of brain noradrenaline metabolites was unremarkable in the absence of a panic attack. Perhaps the locus coeruleus and its noradrenergic projections are activated during a panic attack, but we were unable to test that.

The efficacy of SSRI drugs in MDD and PD has generated a *serotonin deficiency* hypothesis of both disorders (2), with serotonin reuptake-blockade after CNS release being imagined to correct a shortfall in neuronal serotonin release. This *heuristic* is just that, an imagined formulation of CNS neurotransmitter abnormality without definitive sustaining scientific evidence. In reality, it is difficult to test for serotonin transmitter mechanisms of psychiatric illness because several brain serotonergic neuronal systems have actions unrelated to neuropsychiatry, including those linked to feeding and obesity, sunlight exposure, biorhythms and seasonality (15, 23–25). In the testing we did of brain serotonin turnover in MDD and PD, we attempted to avoid confounding by these extraneous factors though selection of the healthy comparator groups such as to be matched with the patient groups for body mass index (BMI) and sunlight hours exposure on the catheter testing day.

Good matching for BMI and sunlight hours on the testing day was actually achieved. The ~4-fold increase in brain 5HIAA overflow in PD, as we have noted previously (12) was unrelated to a genetic polymorphism impairing neuronal serotonin reuptake, was proportional to clinical indices of PD severity, and during SSRI dosing was often normalized in concert with patient clinical improvement (12). The reported finding of reduced capacity for serotonin ligand binding to brain serotonin receptors in PD (29, 30) is in harmony with our own observation, with presumed excess serotonin release in PD competing with the scanning ligand for receptor binding sites. Applying scanning methodology in parallel with the jugular venous technique would be instructive. PET and SPECT studies, ideally utilizing both 5-HT_1A_ and 5-HT_1B_ serotonergic ligands, should in the future provide regional measures of receptor occupancy which would be complementary to the measures of brain 5HIAA overflow based on jugular venous sampling. Scanning methodology should provide brain topography information further helping in the differentiation of brain serotonin neurons related to anxiety from those serotonergic neuronal pools related to seasonality and to feeding/satiety.

The finding of increased brain 5HIAA overflow in panic disorder is contrary to the commonly held view that brain serotonin release is diminished, but does fit well with some clinical and experimental observations. Common clinical experience is that these drugs can cause agitation and increased frequency of panic attacks as treatment is started (31). Clinical improvement occurs typically only after a few weeks of dosing. As panic disorder can be interpreted by our results as being characterized by increased brain neuronal serotonin release, this early clinical deterioration is as would be expected from a drug class increasing the synaptic concentration of serotonin. The phase of clinical improvement seen later possibly coincides with inhibition of firing of serotonergic neurons, by an extrasynaptic inhibitory action of serotonin on the neuronal cell body, although this interpretation does remain speculative.

A striking parallel was seen in MDD patients, where estimated brain 5HIAA overflow was increased ~4-fold, and typically normalized when illness remission was achieved with SSRI dosing. In MDD, however, depression severity was unrelated to this. Can we be sure that 5-HIAA, the serotonin metabolite we measure in the brain venous effluent, truly has a brain neuronal origin? Experimental studies indicate there is some potential for serotonin released by platelets into plasma to be taken up and metabolized to 5-HIAA in both sympathetic nerves and vascular endothelium (32, 33), including within the cerebrovascular circulation. Human vascular preparations, however, have only minimal capacity to take up serotonin (34), so a brain neuronal origin of the excess 5-HIAA appears assured.

It is important to note that we assessed brain serotonin turnover from the measurement of brain 5HIAA overflow, and did not directly measure serotonin release. Serotonin turnover approximately equates with serotonin synthesis rate, which we inferred from the rate of 5-HIAA efflux from the brain, given that 5-HIAA is the predominant metabolite of the neurotransmitter (33). A substantial proportion of serotonin turnover derives from intra-neuronal transmitter vesicular escape, with subsequent metabolism by monoamine oxidase (11). Accordingly, serotonin turnover markedly exceeds the serotonin release rate. In the absence of a major disturbance in the stability of neuronal vesicular storage in MDD and PD, however, the estimated serotonin turnover increase in PD and MDD provides some information on neuronal serotonin release. Serotonin transporter genotyping demonstrates that the brain 5HIAA change we document in PD is not due to impaired serotonin reuptake (10), but most likely results from increased firing in midbrain raphe serotonergic neurons projecting to and releasing serotonin in the cerebral cortex and subcortical brain regions. Studies in experimental animals have demonstrated the importance of serotonergic raphe nuclei in anxiety responses (34). Beyond this probability, understanding the core significances of our findings does remain difficult. Why is brain 5HIAA overflow so increased in MDD also? MDD and PD are two distinct psychiatric conditions although, it is noted often mutually comorbid? And is the reduction in brain 5HIAA overflow during SSRI dosing the mechanism of therapeutic drug action?

MDD and PD are both characterized by sympathetic nervous system activation, persistent sympathetic activation in MDD (27, 28) and episodic sympathetic activation in PD, during panic attacks (35, 36). Chronic or recurring human sympathetic activation can be a lethality factor (37). A specific and noxious “toxic trifecta” of *activation of the cardiac sympathetic outflow*, coupled with *impaired sympathetic neural reuptake of noradrenaline*, and *firing of single sympathetic fibers in multiple salvos* within a cardiac cycle (38) exposes the heart to excessive noradrenaline concentrations and is demonstrably harmful (34). Both MDD and PD share these adverse sympathetic nervous system characteristics (28, 35–37). It is pertinent to emphasize this, in the present volume addressing mind-heart linkage., and further, to note that increased clinical cardiovascular risk is present in MDD (38) and PD (39, 40). In PD this is perhaps unexpected, but is proven. Could the CNS serotonergic activation we infer in MDD and PD, perhaps in the midbrain raphe, be the driver of this sympathetic activation? This is unlikely, as we have previously demonstrated that the dominant mechanism of activation of the human central sympathetic outflow in other pathophysiological contexts (hypertension, heart failure) is noradrenaline release in the hypothalamus and amygdala, from the projections of brainstem noradrenergic neurons (8, 41).

## Author Contributions

ME conceived the depressive illness and panic disorder studies, was primary supervisor of MA and DB in their thesis work, managed and funded the laboratory performing the research, and contributed to study design and data analysis. MA led the panic disorder project, as her primary Ph.D., thesis study, contributed to all levels of the panic disorder component of the work, including patient recruitment and diagnosis, and contributed to study design and data analysis. DB led the depressive illness project as his primary Ph.D., thesis study, contributed to all levels of the depressive illness component of the work, including patient recruitment and diagnosis, and contributed to study design and data analysis. GJ and DK led the invasive study day and performed internal jugular catheterizations. LG contributed to neurochemical assays and interpretation of results. RS contributed to study design and diagnostic criteria. GL developed the brain monoamine turnover methodology and contributed to neurochemical assays. All authors contributed to interpretation of results and review of manuscript first draft.

## Funding

This work was supported in part by the Victorian Government's Operational Infrastructure Support Program, and by National Health and Medical Research Council of Australia project grant funding, and Fellowship funding (ME, DK, and GL).

## Conflict of Interest

The authors declare that the research was conducted in the absence of any commercial or financial relationships that could be construed as a potential conflict of interest.

## Publisher's Note

All claims expressed in this article are solely those of the authors and do not necessarily represent those of their affiliated organizations, or those of the publisher, the editors and the reviewers. Any product that may be evaluated in this article, or claim that may be made by its manufacturer, is not guaranteed or endorsed by the publisher.
